# Percutaneous radiofrequency treatment of the gasserian ganglion for trigeminal neuralgia complicated by trochlear nerve palsy: a case report

**DOI:** 10.1136/rapm-2020-102285

**Published:** 2021-05-26

**Authors:** Pascal SH Smulders, Michel AMB Terheggen, José W Geurts, Jan Willem Kallewaard

**Affiliations:** 1 Department of Anesthesiology and Pain Medicine, Amsterdam UMC location AMC, Amsterdam, The Netherlands; 2 Department of Anesthesiology and Pain Medicine, Rijnstate, Arnhem, The Netherlands

**Keywords:** facial pain, pain management, postoperative complications, treatment outcome

## Abstract

**Background:**

Trigeminal neuralgia (TN) has the highest incidence of disorders causing facial pain. TN is provoked by benign stimuli, like shaving, leading to severe, short-lasting pain. Patients are initially treated using antiepileptic drugs; however, multiple invasive options are available when conservative treatment proves insufficient. Percutaneous radiofrequency treatment of the trigeminal, or gasserian, ganglion (RF-G) is a procedure regularly used in refractory patients with comorbidities. RF-G involves complex needle maneuvering to perform selective radiofrequency heat treatment of the affected divisions. We present a unique case of cranial nerve 4 (CN4) paralysis after RF-G.

**Case presentation:**

A male patient in his 60s presented with sharp left-sided facial pain and was diagnosed with TN, attributed to the maxillary and mandibular divisions. MRI showed a vascular loop of the anterior inferior cerebellar artery without interference of the trigeminal complex. The patient opted for RF-G after inadequate conservative therapy. The procedure was performed by an experienced pain physician and guided by live fluoroscopy. The patient was discharged without problems but examined the following day for double vision. Postprocedural MRI showed enhanced signaling between the trigeminal complex and the brainstem. Palsy of CN4 was identified by a neurologist, and spontaneous recovery followed 5 months after the procedure.

**Conclusions:**

Mention of postprocedural diplopia in guidelines is brief, and the exact incidence remains unknown. Different mechanisms for cranial nerve (CN) palsy have been postulated: incorrect technique, anatomical variations, and secondary heat injury. We observed postprocedural hemorrhage and hypothesized that bleeding might be a contributing factor in injury of CNs after RF-G.

## Introduction

Chronic facial pain can be the result of a large number of different conditions. Trigeminal neuralgia (TN) is the disorder with the highest incidence within this group.^1^ TN is characterized by a severe, short-lasting paroxysmal pain, elicited by stimuli like shaving, cold or eating. The International Headache Society recognizes three distinct subtypes of TN: classical, secondary and idiopathic. Classical TN refers to the subset of patients in whom imaging reveals a neurovascular conflict or vascular compression of the trigeminal nerve, while secondary TN is caused by another illness, like multiple sclerosis. Etiology of idiopathic TN is unidentified.^2^ Although the exact pathophysiology of TN remains unclear, morphological changes to the trigeminal nerve root are observed in a significant proportion of patients. It is speculated that these changes result in focal demyelination, which then leads to unwarranted action potential generation and facial pain.^3^ Incidence is estimated at 12.6 per 100 000 per year in the general population and increases with age. It affects men to women in a ratio of 1.0:2.44.^1^

TN is an often debilitating disease that has a substantial impact on quality of life.^4^ Most patients are treated with antiepileptic drugs, when necessary in combination with other conservative treatments such as psychological therapy. When conservative strategies prove insufficient, a wide palette of interventional procedures are optional as second-line therapy.

Open microvascular decompression (MVD) is associated with the highest rates of morbidity and mortality, and is usually reserved for younger, healthy patients with confirmed neurovascular conflict. Refractory patients with comorbidities can be accepted for less invasive treatment, such as percutaneous radiofrequency treatment of the gasserian ganglion (RF-G) or gamma-knife radiosurgery. Level of evidence for efficacy of these procedures is considered weak to very weak.^5^

**Figure 1:**
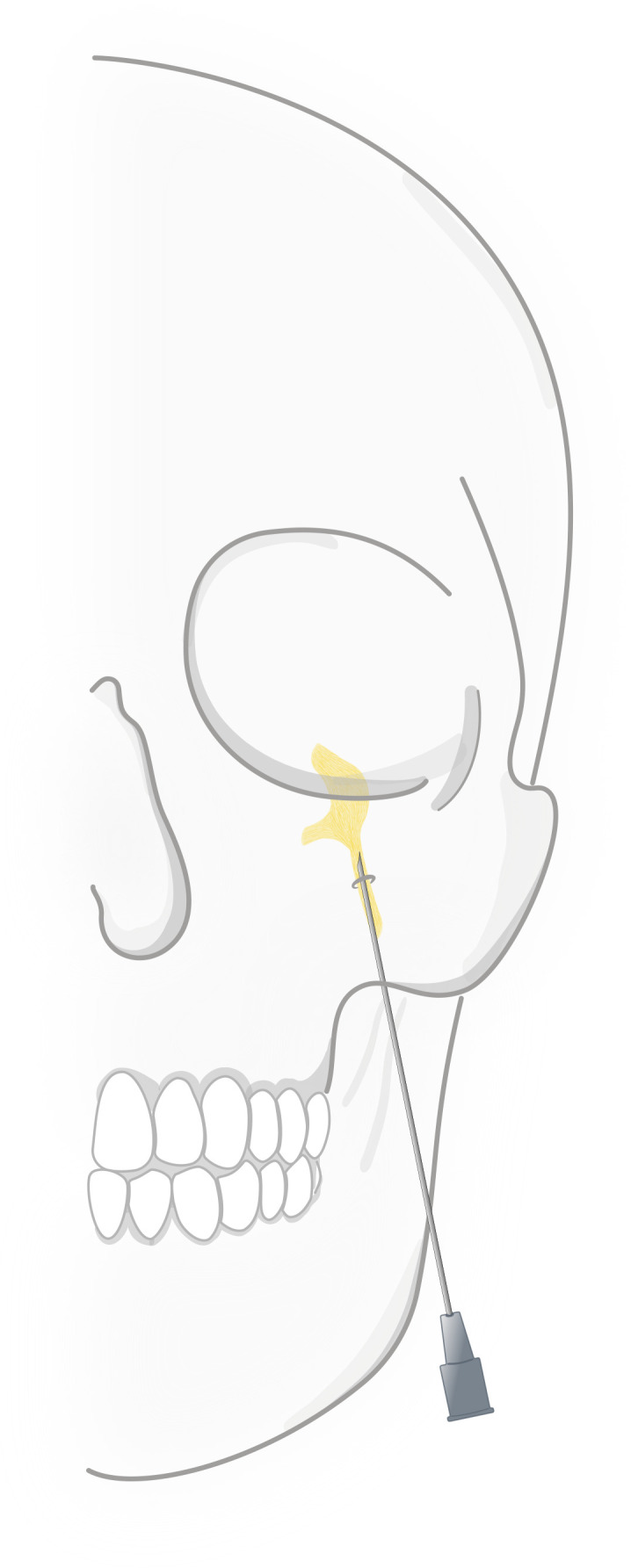
Steep path of the needle tip as it is inserted in the cheek and passes behind the zygomatic bone and through the foramen ovale to the trigeminal ganglion.

RF-G is a technique that allows for selective lesioning of trigeminal nerve divisions. This procedure, first performed by Sweet in 1974, uses real-time fluoroscopy imaging to aid the maneuvering of the needle tip through the foramen ovale to Meckel’s cave and the trigeminal ganglion (schematically illustrated in figure 1).^6^ On-site RF-G targets the trigeminal or gasserian ganglion by radiofrequency-induced heat.

RF-G is generally considered safe and complications are few. In a cohort of 1600 patients, Kanpolat *et al* reported 225 patients with complications after 2138 RF-G procedures. Diminished corneal reflex was observed in 91 patients (5.7%), masseter dysfunction in 66 (4.1%), anesthesia dolorosa and carotid puncture both in 12 patients (each 0.75%), keratitis in 10 patients (0.6%), cerebrospinal fluid leakage in 2 (0.13%) and meningitis in 1 (0.06%). Transient palsy of a cranial nerve (CN) was present in 12 patients (0.75%) (CN3 1 patient, CN6 11 patients). Permanent damage to CN6 was reported for two patients (0.13%).^7^

CN palsy is a rare complication of RF-G, and the majority of studies citing CN palsies date back decades ago. CN damage after invasive treatment for TN seems not to be linked to one particular CN, and literature is not conclusive to which CN is most prone to injury. We present a unique case of isolated transient trochlear nerve (cranial nerve 4 (CN4)) paralysis after percutaneous radiofrequency treatment of the gasserian ganglion for TN.

## Case report

A male patient in his 60s presented to the outpatient pain clinic with sharp left-sided facial pain. The pain was provoked by washing, chewing, and the application of his continuous positive airway pressure mask. TN was diagnosed in accordance with the International Classification of Headache Disorders.^2^ Symptoms were attributed to the maxillary and mandibular divisions of the trigeminal nerve. Medical history included obstructive sleep apnea, depression, and one incident of probable transient ischemic attack of the brain. Treatment with carbamazepine provided adequate pain relief, but the patient was afraid of consequences to his driving ability with escalating dosage and was hesitant to long-term use.

MRI showed a vascular loop of the anterior inferior cerebellar artery (AICA) without compression or displacement of the left trigeminal complex, and a prominent AICA on the right side. The other (trigeminal) anatomy showed no abnormalities. RF-G of the maxillary and mandibular divisions was scheduled.

The procedure was performed in a specialized outpatient interventional pain center by an experienced pain physician who performs an annual minimum of 20 RF-G procedures. Sedation was administered by a certified nurse sedation practitioner specialist using propofol. Maneuvering of the 22G cannula (SMK Radiofrequency Cannula—straight 10 cm with 2 mm tip; St. Jude Medical) was assisted by live fluoroscopy and executed in sterile fashion. Four positions of the cannula were tested under fluoroscopic guidance before acceptable sensory stimulation of the maxillary and mandibular divisions was attained. The final, and most cephalad, position of the needle tip was slightly past the angle formed by the petrosal ridge of the temporal bone and the clivus. Stimulation was performed awake to confirm correct needle positioning. As the ultimate sensory threshold of 0.3 V was on the edge of the target range, radiofrequency lesioning was performed directly at 70°C during 60 s, instead of starting at 60°C. Motor response was absent at 2.0 V. The sensory threshold rose from 0.3 to 0.8 V after the lesion was applied, and no subsequent lesions were made. Postoperative corneal reflex was intact; vision and facial sensibility were normal.

**Figure 2:**
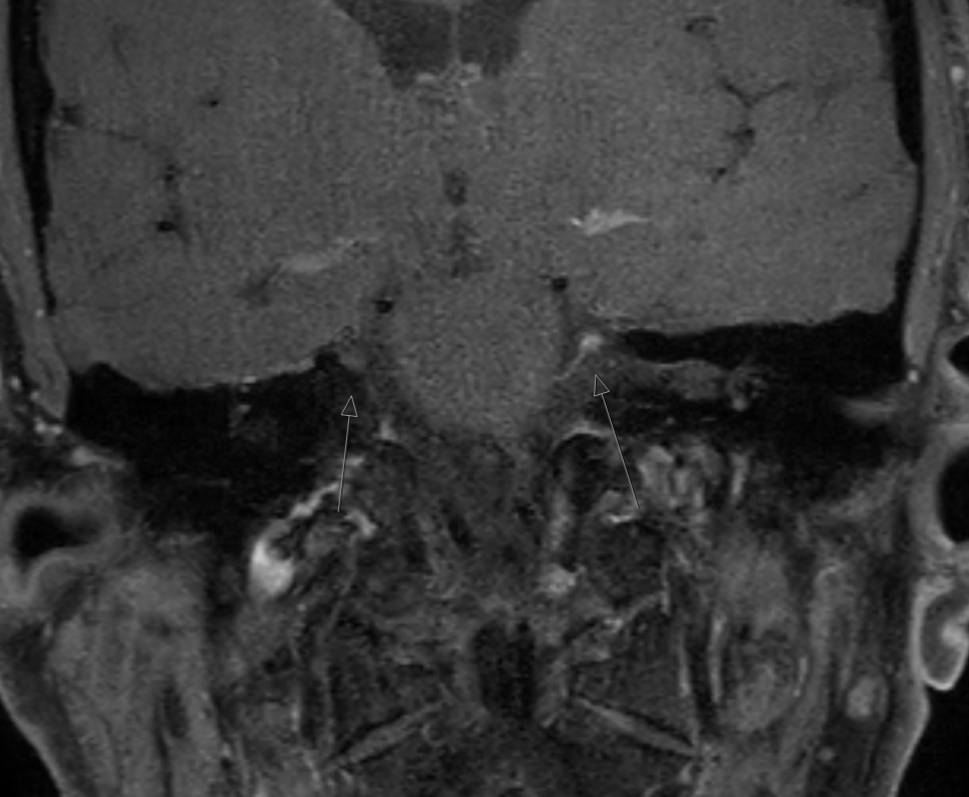
Postprocedural MRI of the brain showing enhanced signaling on the procedural side. The arrows point to the location of enhanced signalling (ie the hematoma) of the MRI.

The day following the procedure, the patient complained of double vision and was examined at the emergency department by a neurologist. Diplopia was most pronounced when looking down. Monocular eyesight was normal, facial pain was absent. Postprocedural MRI of the brain showed enhanced signaling on the procedural side between the trigeminal ganglion and the brainstem (figure 2), and (asymptomatic) sinusitis of the maxillary and sphenoid sinus. Ophthalmic consultation established a restriction in function of the left superior oblique muscle, which is innervated by CN4. Prism correction proved unhelpful and otolaryngology examination gave no new insights. Trochlear nerve function spontaneously recovered to normal 5 months after the procedure.

The patient approved publication of the case history.

## Discussion

Large, well-established trials on outcome after treatment for TN are few. As such, most data on complication risk are derived from a select number of decades old cohort studies. The incidence of CN paralysis after RF-G is reported to be between 0.29% and 7.7%, with more recent studies citing numbers at the lower end of this range.^7–12^ These studies are regularly referenced in guidelines, but mention of postprocedural diplopia is mostly lacking. Although the incidence of diplopia after RF-G remains unclear, clinical experience confirms that occurrence of CN palsy after RF-G is exceedingly rare.

A handful of case reports concerning CN palsy after treatment for TN have been published over the last decennia. Treatment modalities for these patients span all procedures, except for gamma-knife surgery. Multiple authors report isolated transient CN4 or CN6 palsy after (repeated) percutaneous balloon compression, RF-G, or MVD.^13–18^ One study documents a patient with permanent trochlear nerve damage and corresponding diplopia after RF-G.^19^ Subarachnoid hemorrhage after attempted RF-G, followed by — among other symptoms — multiple bilateral CN paralysis, has also been reported.^20^

Preprocedural imaging has become standard practice, ensuring clinicians of knowledge of a patient’s anatomy. Preprocedural MRI has an important role in finding the cause of TN and may detect anatomical variations important to consider when planning invasive procedures. However, a certain level of imprecision or ambiguity is inherent to imaging studies as the trigeminal complex and Meckel’s cave are relatively small anatomical structures and can usually only be partially visualized. The postprocedural diagnosis of an arteriovenous malformation in a patient with CN4 palsy after percutaneous balloon compression demonstrates this imprecision and illustrates that preprocedural MRI is unable to prevent all cases of CN palsy.^21^

The trochlear nerve has the longest intracranial course of all CNs and is the only one to originate from the dorsal midbrain. It decussates in the superior medullary velum, transverses the brain and enters the cavernous sinus to terminate on the superior oblique muscle.^22 23^ The decussating path of the trochlear nerve allowed the conclusion that the trochlear nerve, not the trochlear nucleus, was injured in this case history. The cavernous sinus is a dural envelope that contains not only CN4, but also CN3 and CN6.^23^ Its boneless makeup and close relation to Meckel’s cave may play a role in the mechanism underlying CN paralysis after RF-G.

Various mechanisms for CN injury have been postulated, of which the most prominent is incorrect procedural technique. The proximity of the trigeminal complex to the different CNs warrants extreme caution, as minute displacement of the cannula in either depth or direction may lead to complications. The application of fluoroscopy enables real-time needle visualization and is thought to be crucial to minimize the incidence of adverse events.

Different authors hypothesize that the origin of CN palsy is equipment-related, that is, insertion too far into Meckel’s cave, pertaining to cannula design, or overinflation in the setting of balloon compression.^16 17 19 24 25^ Other mentioned causes of CN damage are anatomical variations and secondary heat injury originating from the outward radiation of thermal energy from the target site.^16 18 19 21^

We propose a novel explanation and hypothesize that procedure-related hemorrhage might lead to compression and injury of CNs. We found enhanced postprocedural magnetic resonance signaling in this patient and presume this to be a reflection of hematoma. Hemorrhage might be the causative mechanism for CN4 palsy in this case, but other explanations could be (undetected) anatomical variations or secondary heat injury.

In conclusion, trochlear nerve palsy resulting in diplopia is a rare, generally transient complication of percutaneous radiofrequency treatment of the gasserian ganglion. Preprocedural imaging is essential for finding the origin of TN and contributes to risk assessment when planning invasive treatment techniques, while awake stimulation and careful needle maneuvering under fluoroscopic guidance are needed for correct application of RF-G.
